# Persistent Exposure to *Fusobacterium nucleatum* Triggers Chemokine/Cytokine Release and Inhibits the Proliferation and Osteogenic Differentiation Capabilities of Human Gingiva-Derived Mesenchymal Stem Cells

**DOI:** 10.3389/fcimb.2019.00429

**Published:** 2019-12-17

**Authors:** Wenyan Kang, Xiaoli Ji, Xiujun Zhang, Di Tang, Qiang Feng

**Affiliations:** ^1^Department of Human Microbiome, School and Hospital of Stomatology, Shandong University and Shandong Provincial Key Laboratory of Oral Tissue Regeneration and Shandong Engineering Laboratory for Dental Materials and Oral Tissue Regeneration, Jinan, China; ^2^Department of Periodontology, School of Stomatology, Shandong University, Jinan, China; ^3^Department of Oral Medicine, School of Stomatology, Shandong University, Jinan, China; ^4^State Key Laboratory of Microbial Technology, Shandong University, Qingdao, China

**Keywords:** *Fusobacterium nucleatum*, gingival mesenchymal stem cells, cell proliferation, inflammatory cytokines, osteogenesis differentiation

## Abstract

*Fusobacterium nucleatum* is one of the most frequent pathogenic bacteria causing periodontitis. The direct effect of *Fusobacterium nucleatum* (*F. nucleatum*) on oral stem cells has rarely been reported. In this study, we aimed to evaluate how gingiva-derived mesenchymal stem cells (GMSCs) respond to a direct challenge with *F. nucleatum*. GMSCs were isolated by the limiting dilution method and exposed to *F. nucleatum* at various multiplicities of infection (MOIs; *F. nucleatum*:cell ratios of 10:1, 50:1, and 100:1) for 24 h to 4 weeks. Our results indicated that *F. nucleatum* significantly inhibited cell proliferation in a dose-dependent manner and promoted cell migration and the release of chemokines/cytokines, such as CCL2, CXCL1, and IL-6. Additionally, *F. nucleatum* inhibited GMSC osteogenic differentiation partly by decreasing alkaline phosphatase (ALP) activity, mineralized nodule formation, and osteogenesis-related gene and protein expression. RNA-sequencing analyses indicated that *F. nucleatum* time-dependently activated cellular signaling pathways during the process of osteogenic differentiation. A total of 64 cell differentiation-related genes were found to be differentially expressed between non-infected and *F. nucleatum*-infected GMSCs at 3, 7, 14, and 21 d. Intriguingly, we discovered that the 64 cell differentiation-related differentially expressed genes (DEGs) were significantly enriched in cancer-related pathways, such as bone cancer, osteosarcoma and bone marrow cancer, which provides new insight into tumorigenesis during the process of GMSC osteogenic differentiation. In conclusion, this study demonstrates that persistent exposure to *F. nucleatum* promotes cell migration and chemokine/cytokine release and inhibits the proliferation and osteogenic differentiation of GMSCs. Our study provides a novel and long-time bacteria-cell co-culture *in vitro* model and makes a foundation for the future mechanistic studies of GMSCs under *F. nucleatum* infection.

## Introduction

Oral microorganisms are abundant and play key roles in maintaining oral health; periodontitis is a typical chronic infectious disease caused by increased oral pathogenic bacterium (Paster and Dewhirst, 2009). Groups of oral pathogens contribute to the occurrence of periodontitis; however, their pathogenesis effects in periodontitis have not been fully illuminated. *Fusobacterium nucleatum* (*F. nucleatum*) is a common anaerobe in the oral cavity and has been frequently detected with high abundance in periodontitis patients (Wang et al., 2015). *F. nucleatum* is an opportunistic pathogen closely associated with the occurrence and development of periodontitis, and its abundance is positively associated with the periodontal pocket depth (Papapanou et al., 1993; Moore and Moore, 1994; Socransky et al., 1998). Studies have shown that *F. nucleatum* possesses versatile adhesion properties and can co-aggregate with all other strains of oral bacteria (Brook and Walker, 1986; Kinder and Holt, 1993). The dental biofilm, which is composed of a large number of bacteria has been identified as the most difficult therapeutic target for periodontitis (Moore and Moore, 1994; Socransky and Haffajee, 2002). *F. nucleatum* has its unique pathogenic potential and is dominant in the dental biofilm at a later stage, which play critical roles in the destruction of periodontal supporting tissues (Moore and Moore, 1994; Ebersole et al., 1995). However, if *F. nucleatum* could directly destruct the periodontal supporting tissues and the potential pathogenic mechanism have not been fully elucidated.

The *F. nucleatum* in periodontal pockets can easily invade the host tissue and interact with the host cells (Jeng et al., 1999). *F. nucleatum* has been reported to exert pathogenic effects by producing a group of virulence factors and toxic metabolites, such as lipopolysaccharide, porin, butyrate and propionate ammonia (Takada et al., 1988; Rogers et al., 1991; Bolstad et al., 1996). In peripheral blood mononuclear and polymorphonuclear cells, *F. nucleatum* induces apoptosis by activating caspases and then plays an immunosuppressive role in host cells (Jewett et al., 2000). In gingival epithelial cells (GECs), *F. nucleatum* can activate innate immune responses and promote the secretion of proinflammatory cytokines and matrix metalloproteinases (MMPs), which finally play crucial roles in the destruction of the periodontal supporting tissues (Gursoy et al., 2008, 2012). *F. nucleatum* can promote the secretion of interleukin (IL)-1β in GECs by activating the NLRP3 inflammasome (Bui et al., 2016).

The gingival epithelium is recognized as the first barrier against oral infection (Ji et al., 2009; Signat et al., 2011). However, *F. nucleatum* can break through the epithelial barrier and penetrate into the gingival connective tissues in patients with severe periodontitis (Signat et al., 2011). It has been reported that *F. nucleatum* can induce the release of interleukin-6 in gingival fibroblasts and significantly inhibit the cell proliferation (Bartold et al., 1991; Rossano et al., 1993). Recently, researchers have isolated a new type of mesenchymal stem cell from human gingival connective tissue, namely, gingival tissue-derived mesenchymal stem cells (GMSCs) (Fournier et al., 2010; Mitrano et al., 2010). GMSCs possess normal karyotype and telomerase activity and exhibit a stable phenotype in long-term cultures; furthermore, these cells have self-renewal, multipotent differentiation, and immunomodulatory abilities, which have attracted increasing attention from scholars (Fournier et al., 2010; Mitrano et al., 2010; Tomar et al., 2010; Tang et al., 2011). Given their accessibility and relative abundance in gingival tissues, GMSCs have a bright future in cell-based tissue regenerative medicine for the treatment of human dental diseases and the improvement of human health (Zhao et al., 2015). In previous studies, GMSCs have been widely used in nerve regeneration and bone tissue regeneration (Wang et al., 2011; Mao et al., 2019). In addition, Li et al. reported that the inflammatory microenvironment could induce GMSCs differentiation toward a profibrotic phenotype and then lead to inflammatory gingiva hyperplasia, which is detrimental for GMSC-based therapy (Li et al., 2013). However, the detailed biological characteristic changes of GMSCs in the inflammatory environment have not been reported thus far, and the effect of *F. nucleatum* on GMSCs has not been previously examined. In this study, we aimed to explore how GMSCs respond to a direct challenge with *F. nucleatum* by evaluating the proliferation, migration, and inflammatory cytokine production and the osteogenic differentiation ability in *F. nucleatum* infected GMSCs. In addition, we performed a comprehensive gene expression profile analysis of *F. nucleatum* infected GMSCs during the process of osteogenic differentiation. Our study provides a novel and long-time bacteria-cell co-culture *in vitro* model and performs comprehensive biological characteristic changes of GMSCs under time-series *F. nucleatum* infection, which makes a foundation for the future mechanistic studies of GMSCs under *F. nucleatum* infection.

## Materials and Methods

### Human Subjects and Ethical Statements

This study was approved by the Medical Ethical Committee of the Stomatology School, Shandong University (Protocol Number: 20170101). Three healthy individuals, aged from 20 to 25 years, who underwent impacted tooth extraction at the Stomatology Hospital of Shandong Province, were recruited. All individuals were informed about the research project and signed the informed consent according to the Helsinki Declaration of 1975.

### Bacterial Strains, Cell Cultures, and Identification

*Fusobacterium nucleatum* subsp. nucleatum ATCC 25586 was provided by Shandong Provincial Key Laboratory of Oral Tissue Regeneration (Jinan, China). Stock cultures were routinely propagated in brain-heart infusion (BHI, Haibo, Qingdao, China) broth supplemented with 5 μg/ml hemin and 1 μg/ml menadione in an anaerobic atmosphere with 85% N_2_, 5% H_2_, and 10% CO_2_ for 24 h in a constant temperature incubator at 37°C. *F. nucleatum* was tested and verified by polymerase chain reaction (PCR) assay and 16S rRNA gene sequencing. The primers used are shown in Supplementary Table 1. *F. nucleatum* cultured in the logarithmic growth phase was harvested by centrifugation at 4,500 rpm for 5 min and washed three times with phosphate buffered saline (PBS, HyClone, Logan, UT, USA). The bacterial concentration was spectrophotometrically standardized to optical density (OD) value at 600 nm = 1 *F. nucleatum*, corresponding to 10^8^ bacteria/ml. Finally, the cells were suspended in Dulbecco's modified Eagle's medium (DMEM, HyClone) for subsequent studies.

### Cell Isolation and Culture

The excised gingival tissues were immediately immersed in DMEM with 5% antibiotics (100 mg/ml penicillin, 100 mg/ml streptomycin, Sigma Aldrich, St Louis, MO, USA) and quickly transferred to the laboratory. Then the free gingival tissue samples were washed, and the epithelium was removed and minced into small fragments of ~1–3 mm^2^ and digested for 2 h at 37°C by 3 mg/ml collagenase I (Sigma Aldrich) and 4 mg/mL dispase II (Invitrogen, Carlsbad, CA, USA). To obtain GMSCs, the single-cell suspension was filtered through a 70 μm cell strainer and seeded into 10 cm Petri dishes at a density of 60 cells per cm^2^ according to a previous study (Ge et al., 2012). The primary GMSCs were cultured with DMEM containing 20% fetal bovine serum (FBS, BioInd, Kibbutz, Israel) at 37°C in a humidified atmosphere of 5% CO_2_. Cells were fed fresh medium every 3 days until the cell monolayer reached 80–90% confluence. GMSCs were trypsinized and passaged at a dilution ratio of 1:3 to expand the culture in 10% FBS DMEM medium without antibiotics. The fourth passage cells were used for subsequent experiments.

### Colony Formation and Flow Cytometric Analysis

GMSCs were seeded in six-well-plates at a density of 1 × 10^3^ cells/well and cultured with DMEM containing 10% FBS; and the cells were fed fresh medium every 3 days. After 7 d, the cells were fixed in 4% paraformaldehyde (Sigma Aldrich) for 30 min and stained with crystal violet (Solarbio, Beijing, China) for 5 min. The total number of cell colonies was photographed and counted under a high-power microscope (Olympus, Tokyo, Japan). Groups of fifty or more adherent cells derived from the same mother cell were assessed as a colony. For the phenotypic characterization of stem cells, GMSCs infected with or without *F. nucleatum* at the multiplicity of infection (MOI) of 100 at 3 d were collected and resuspended in blocking buffer with 1% immunoglobulins G and M for 30 min and then incubated with fluorescein-conjugated mouse monoclonal antibodies (10 μg/ml) specific for human CD29, CD44, CD73, and CD45 (Becton Dickinson Biosciences, NY, USA) for 30 min on ice. After being washed, the cells were fixed in PBS and subjected to flow cytometric analyses.

### Cell Morphology and Proliferation Assays

GMSCs were seeded in 6-well (1 × 10^5^ cells/well) plates in growth medium with 10% FBS and no antibiotics. Cells were left untreated or incubated with *F. nucleatum* at multiplicities of infection (MOIs) of 10, 50, and 100 (*F. nucleatum*:cell ratio of 10:1, 50:1, 100:1) from 1 to 7 d, and cells were fed fresh medium every day. The cell morphology was detected by crystal violet (Solarbio) staining. Cells were fixed in 4% paraformaldehyde for 30 min and stained with crystal violet (Solarbio) for 30 min. The cells were observed immediately under the microscope and photographed (Olympus). Cell proliferation was detected by counting the cell numbers through an automated cell counter (Countstar, Shanghai, China). Briefly, cells infected with or without *F. nucleatum* were trypsinized, collected in a 1.5 ml microcentrifuge tube and centrifuged for 5 min at 1,000 rpm. After being washed twice with PBS, cells were resuspended in 1 ml of PBS and mixed thoroughly. Then, 20 μl of cell resuspension solution was added to the counting chamber and detected by a cell counter (Countstar). Cell proliferation rate was detected by the 5-ethynyl-20-deoxyuridine (EdU) labeling assay according to the instructions of an EdU Apollo DNA *in vitro* kit (RIBOBIO, Guangzhou, China).

### Cell Migration Assay

The effect of *F. nucleatum* on GMSCs migrations was evaluated by a transwell chamber with an 8.0 μM pore size (Corning, Corning, NY, USA). Approximately 5 × 10^4^ cells cultured in complete medium with no antibiotics were seeded in the upper chamber, and the lower plates were filled with 500 μl of 10% FBS containing medium with or without *F. nucleatum* at MOIs of 10, 50, and 100. Medium containing 10% FBS was used as a negative control (NC). The chambers were incubated for 20 h at 37°C. The cells that migrated through the membrane were fixed in 4% paraformaldehyde and stained with 0.1% crystal violet. Six high-power microscopic fields (×200) per filter were randomly selected by blind evaluation and the number of cells that had migrated into the undersurface of the membrane was counted.

### Enzyme-Linked Immunosorbent Assay (ELISA)

GMSCs (2 × 10^5^ cells/well) were seeded in 6-well-plates, cultured with 10% FBS DMEM without antibiotics and treated with *F. nucleatum* at MOIs of 10, 50, and 100 for 24, 48, and 72 h. The cell culture supernatants were collected and centrifuged at 12,000 rpm for 5 min at 4°C. The levels of secreted protein IL-6, IL-8, and IL-1β were measured by ELISA (BioLegend, San Diego, CA, USA) according to the manufacturer's instructions. The OD values were measured by a microplate reader (SPECTROstar Nano, BMG Labtech, Offenburg, Germany) at 450 and 570 nm, and the values at 570 nm were subtracted from those at 450 nm in the following data analysis. The experiment was performed in triplicate and repeated three times with cell culture supernatants from 3 different donors.

### Alkaline Phosphatase (ALP) Activity Assay

GMSCs (2 × 10^5^ cells/well) were seeded in 6-well-plates and cultured with osteogenic medium without antibiotics [OM, medium supplemented with 10% FBS, 10^−8^mol/L dexamethasone (Solarbio), 50 mg/L ascorbic acid and 10 mmol/L β-glycerophosphate (Sigma-Aldrich)]. *F. nucleatum* was used to stimulate cells at MOIs of 10, 50, and 100 for 3, 7, 14, and 21 d. The medium and *F. nucleatum* were changed every 3 days. Cells were lysed with 1% TritonX-100 (Solarbio) for 30 min. Cell lysates were collected, and the protein concentration was measured according to a bicinchoninic acid (BCA) protein assay kit (CWBIO, Beijing, China). ALP activity was detected according to the instructions of the assay kit (Nanjing Jiancheng Bioengineering Institute, Nanjing, China) and the absorbance was measured with a microplate reader (SPECTROstar Nano) at a wavelength of 520 nm.

### Alizarin Red Staining

GMSCs (2 × 10^5^ cells/well) were seeded in 6-well-plates and cultured in OM (no antibiotics included) with or without *F. nucleatum* (MOIs of 10, 50, and 100) for 14, 21, and 28 d. The medium and *F. nucleatum* were changed every 3 days. The cells were fixed in 4% paraformaldehyde, and extracellular matrix calcification was estimated by 2% alizarin red S (pH 4.3, Sigma-Aldrich). The relative amount of calcium was quantified with 10% (w/v) cetylpyridinium chloride (CPC, Solarbio) and 10 mM sodium phosphate solution. The absorbance was measured at a wavelength of 562 nm.

### RNA Isolation and Quantitative Real-Time PCR (qRT-PCR)

GMSCs (2 × 10^5^ cells/well) were seeded in 6-well-plates and cultured with DMEM without antibiotics and then infected with *F. nucleatum* (MOIs of 10, 50, and 100) for different lengths of times. Total RNA was extracted with TRIzol (CWBIO) and the mRNA concentration was determined using an ultramicro spectrophotometer (Thermo, Waltham, MA, USA). One microgram of mRNA was reverse-transcribed to cDNA using a cDNA synthesis reagent kit (CWBIO). qRT-PCR was performed with UltraSYBR (CWBIO) on a LightCycler 96 Real-Time PCR System (Roche, Basel, Switzerland) in triplicate. Data were analyzed using the 2^−ΔΔ^Ct method. The sequences of the primers used for amplification are shown in Supplementary Table 2.

### Western Blot Assay

GMSCs (2 × 10^5^ cells/well) seeded in 6-well-plates-were infected with *F. nucleatum* (MOIs of 10, 50, and 100) for 3, 7, 14, and 21 d. The medium and *F. nucleatum* were changed every 3 days. Cells were harvested with RIPA lysis buffer containing 1% protease inhibitors (CWBIO). Protein concentrations were measured by a BCA protein assay and 20 μg/lane of protein was separated by 10% sodium dodecyl sulfate-polyacrylamide gel electrophoresis (SDS-PAGE) and transferred to a polyvinylidene fluoride (PVDF) membrane (Millipore, Billerica, MA, USA). The primary antibodies were incubated with the membrane at 4°C overnight at the dilution of 1:1,000–10,000 according to the manufacturer's instructions (Supplementary Table 3) and membranes were then incubated with horseradish peroxidase-conjugated secondary antibodies (1:10,000; Proteintech, Chicago, IN, USA) for 1 h at room temperature. The protein bands were visualized with enhanced chemiluminescence reagents (Millipore) and scanned using an extra sensitive imager (Amersham Imager 600; GE Healthcare Life Sciences, Pittsburgh, PA, USA). Image J 1.44 software (NIH, Bethesda, Maryland, USA) was used to quantify the protein expression levels.

### RNA Sequencing (RNA-seq) Analysis

Twenty-four samples from 3 individuals (for each individual, 4 control samples at 3, 7, 14, and 21 d with no *F. nucleatum* treatment, and 4 experimental samples with *F. nucleatum* infection at an MOI of 100 at the same time point were included) were sequenced for the analysis of gene expression at the whole-genome level at Novogene (Beijing, China) by RNA-seq. Total RNA was isolated, evaluated for quality, reverse-transcribed to cDNA, and sequenced on the Illumina platform. After quality control (QC), clean reads were mapped to a reference genome (GRCh38) via hierarchical indexing for the spliced alignment of transcripts (HISAT v2.0.5) (Kim et al., 2015) and featureCounts (v1.5.0-p3) was used to count the number of reads mapped to each gene (Liao et al., 2014). Then, the fragments per kilobase of exon per million reads mapped (FPKM) of each gene was calculated based on the length of the gene, and the read count mapped to the gene. Based on the gene expression level, DEseq2 was used to detect the differentially expressed genes (DEGs) between control groups and *F. nucleatum*-treated groups by an absolute fold-change in DEGs >2 and an adjusted *P* < 0.01. A Gene Ontology (GO) enrichment analysis of the DEGs was implemented by the clusterProfiler R package, and GO terms with corrected *P* < 0.05 were considered significantly enriched (Young et al., 2010). Pathway enrichment analysis of DEGs was performed based on the Disease Ontology (DO) (Bello et al., 2018), Kyoto Encyclopedia of Genes and Genomes (KEGG) database (Kanehisa and Goto, 2000), Reactome database (Croft et al., 2011), and DisGeNET database (Pinero et al., 2017). The protein-protein interaction network was generated by STRING website with the default parameters (von Mering et al., 2005). The RNA sequencing data have been deposited in the NCBI Gene Expression Omnibus (GEO, http://www.ncbi.nlm.nih.gov/geo/) and are accessible through the GEO series accession number GSE126821.

### Statistical Analysis

The experiment was performed in triplicate and repeated three times. All biological data for validating the effect of *F. nucleatum* on GMSCs were expressed as mean ± standard deviation (SD). First, we checked whether the data confirmed to normal distribution. The normally distributed data were analyzed using GraphPad Prism software (version 6, MacKiev Software, Boston, MA, USA), and differences among more than two groups were analyzed by one-way or two-way ANOVA with Tukey's honestly significant difference (HSD) comparison test. For the data that did not conform to normal distribution, non-parametric analysis with Kruskal-Wallis test was used to analyze the significance of the differences. *P* < 0.05 was considered as statistically significant.

## Results

### Isolation and Characteristics of GMSCs

**Figure 1:**
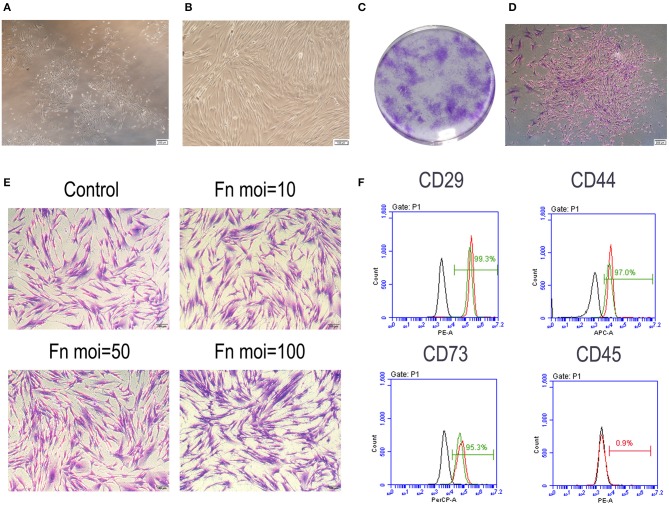
Characteristic of GMSCs. **(A)** Primary GMSCs under low-power magnification; Scale bar: 200 μm. **(B)** The fourth passage GMSCs present a spindle-shaped fibroblast-like morphology. Scale bar: 100 μm. **(C)** Single colonies of GMSCs after 7 d. **(D)** Cell clusters derived from a single GMSC colony. Scale bar: 200 μm. **(E)** Crystal violet staining results of GMSCs morphology without or with *F. nucleatum* infection at MOIs of 10, 50, and 100 at 48 h. Scale bar: 100 μm. **(F)** Flow cytometric analysis of GMSC surface markers, including CD29, CD44, CD73, and CD45. The black line represents the negative control group, the red line represents GMSCs without *F. nucleatum* infection (MOI = 100) and the green line represents the *F. nucleatum*-infected GMSCs.

Primary cultures of single-cell suspensions from human gingival tissues exhibited a spindle-shaped fibroblast-like morphology, which was maintained at the fourth generation (Figures 1A,B). All trials of single cell-derived colony formation were successfully performed (Figure 1C). Cell clusters formed from a single GMSC colony (Figure 1D). A crystal violet staining assay was performed to determine GMSC morphology after *F. nucleatum* infection at MOIs of 10, 50, and 100 for 48 h. The results indicated that *F. nucleatum* induced no significant change in GMSCs morphology (Figure 1E). The surface markers of GMSCs were detected by flow cytometry. GMSCs were uniformly positive (>99%) for the mesenchymal stem cells markers CD29, CD44, and CD73, and negative for the hematopoietic stem cell marker CD45 (<1%), and *F. nucleatum* treatment (MOI = 100) did not influence the expression of surface markers on GMSCs (Figure 1F).

### *F. nucleatum* Inhibited GMSC Proliferation

**Figure 2:**
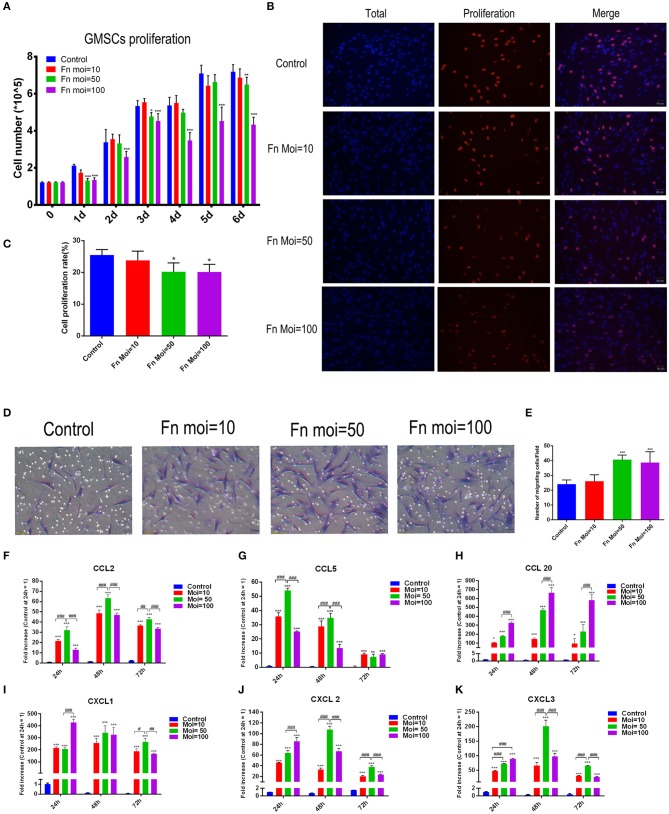
Effects of *F. nucleatum* on GMSC proliferation, migration and chemokine production. **(A)** Number of GMSCs detected by electronic cell counter with or without *F. nucleatum* infection at MOIs of 10, 50, and 100 (*n* = 6). **(B)** EdU assay of GMSCs after *F. nucleatum* infection (MOIs of 0, 10, 50, and 100) at 24 h. Scale bar: 50 μm. **(C)** Cell proliferation rate of GMSCs detected by EdU assay (*n* = 4). **(D)** Migration of human GMSCs infected with *F. nucleatum* at MOIs of 10, 50, and 100. Scale bar: 50 μm. **(E)** Statistical analysis of GMSC migration results. CCL2 **(F)**, CCL5 **(G)**, CCL20 **(H)**, CXCL1 **(I)**, CXCL2 **(J)**, CXCL3 **(K)** chemokine production at the gene expression level in GMSCs co-cultured with *F. nucleatum* (MOIs of 0, 10, 50, and 100) at 24, 48, and 72 h (*n* = 3). All data are shown as the mean ± SD. Statistical analyses were performed by one-way ANOVA with Tukey's multiple-comparison test or non-parametric analysis with Kruskal-Wallis test. *P < 0.05; **P < 0.01; and ***P< 0.001 compared with the control at each time point. ^#^*P* < 0.05, ^*##*^*P* < 0.01, and ^###^*P* < 0.001.

The effect of *F. nucleatum* on GMSCs proliferation were detected by counting the total cell numbers or the new-growth cell numbers in GMSCs infected with or without *F. nucleatum*. A cell counting assay showed that *F. nucleatum* inhibited GMSC proliferation in a dose-dependent manner. Compared with the control group, *F. nucleatum* infection at an MOI of 100 significantly decreased the number of GMSCs from 1 to 6 d at each time point, and the cell proliferation rate was noticeably reduced. GMSCs co-cultured with *F. nucleatum* at an MOI of 10 showed equivalent cell proliferation rates as the control group at each time point. At an MOI of 50, the cell numbers at 1, 3, and 5 d were decreased, whereas they were not significantly different from those in the control group at 2, 4, and 6 d (Figure 2A). The EdU labeling assay demonstrated that *F. nucleatum* infection at an MOI of 50 and 100 significantly decreased the cell proliferation rate of GMSCs at 24 h (Figures 2B,C).

### *F. nucleatum* Promoted GMSC Migration and Chemokine Production

To explore the effect of *F. nucleatum* infection on GMSCs migration, transwell assays were designed and the migration related chemokines were detected. Transwell migration assays indicated that compared with NC groups, GMSC co-cultured groups with *F. nucleatum* at MOIs of 50 and 100 had a significantly enhanced migration capacity (24 ± 1.138 vs. 40 ± 1.282 and 38 ± 3.007 cells/field, *P* < 0.001). *F. nucleatum* at an MOI of 10 also slightly promoted GMSC migration, but the difference was not significant (*P* > 0.05). These results indicated that *F. nucleatum* could enhance GMSC migration in a concentration-dependent manner (Figures 2D,E).

To further explore the mechanism by which *F. nucleatum* promotes cell migration, we detected the gene expression level of chemokines by qRT-PCR after *F. nucleatum* infection, and the results indicated that *F. nucleatum* dose-dependently elevated the expression of the chemokines CCL2, CCL5, CCL20, C-X-C motif chemokine ligand (CXCL)-1, CXCL2, and CXCL3 at 24 h, 48, 72 h (Figures 2F–K). In detail, *F. nucleatum* infection at an MOI of 50 induced the maximum CCL2 gene expression level at 24, 48, and 72 h, whereas the expression of CCL20 reached a peak at 24, 48, and 72 h after *F. nucleatum* infection at an MOI of 100 (Figures 2F,H). For CCL5, the highest mRNA level was induced by *F. nucleatum* at an MOI of 50 at 24 and 48 h; however, at 72 h, no significant difference was observed among the three *F. nucleatum*-infected groups (Figure 2G). The gene expression level of CXCL1 was dramatically elevated by *F. nucleatum* infection at an MOI of 100 at 24 h, whereas the peak appeared at 72 h in the MOI of 50 group (Figure 2I). When cells were co-cultured with *F. nucleatum* for 24 h, the maximum gene expression level of CXCL2 and CXCL3 appeared in the MOI of 100 group, while *F. nucleatum* infection at an MOI of 50 induced the highest mRNA expression of CXCL2 and CXCL3 at 48 and 72 h (Figures 2J,K).

### *F. nucleatum* Promoted Intracellular Enzyme and Inflammatory Cytokine Production in GMSCs

**Figure 3:**
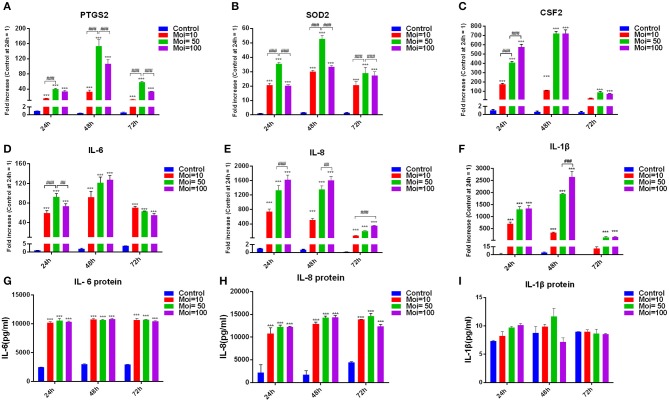
Effects of *F. nucleatum* on GMSC cytokine production. PTGS2 **(A)**, SOD2 **(B)**, CSF2 **(C)**, IL-6 **(D)**, IL-8 **(E)**, and IL-1β **(F)** cytokine production at the gene expression level in GMSCs infected with *F. nucleatum* (MOIs of 0, 10, 50, and 100) at 24, 48, and 72 h (*n* = 3). Protein levels of IL-6 **(G)**, IL-8 **(H)**, and IL-1β **(I)** in GMSCs co-cultured with *F. nucleatum* (MOIs of 0, 10, 50, and 100) at 24, 48, and 72 h (*n* = 3). The histogram represents the mean ± SD. Statistical analyses were performed by two-way ANOVA with Tukey's multiple-comparison test. ***P < 0.001 compared with the control at each time point. ^##^*P* < 0.01 and ^###^*P* < 0.001.

Bacterium infection could directly induce the destruction of periodontal supporting tissues, and to clarify the effect of *F. nucleatum* on altering the inflammation cytokines production in GMSCs, we performed the *in vitro* model of *F. nucleatum* (MOI = 10, 50, and 100) co-cultured with GMSCs at 24, 48, and 72 h. Our results indicated that *F. nucleatum* dose-dependently elevated the expression of intracellular enzyme genes, including prostaglandin-endoperoxide synthase (PTGS)-2 and superoxide dismutase (SOD)-2. The peak expression of PTGS2 and SOD2 appeared after *F. nucleatum* infection at an MOI of 50 for 24, 48, and 72 h (Figures 3A,B). In addition, *F. nucleatum* dose-dependently elevated the production of proinflammatory cytokines, including colony-stimulating factor (CSF)-2, IL-6, IL-8, and IL-1β at the gene level (Figures 3C–F). *F. nucleatum* infection at an MOI of 100 induced the maximum CSF2 gene expression level at 24 h, whereas at 48 and 72 h, CSF2 expression was significantly elevated by *F. nucleatum* infection at MOIs of 10, 50, and 100. No significant difference was observed between the MOI of 50 and MOI of 100 groups (Figure 3C). The gene expression level of IL-6 was increased to the greatest extent by *F. nucleatum* infection at an MOI of 50 at 24 h, whereas no differences existed at 48 h and 72 h with *F. nucleatum* infection at MOIs of 50 and 100 (Figure 3D). For IL-8, the gene expression was significantly increased by *F. nucleatum* infection (MOIs of 10, 50, and 100) at 24, 48, and 72 h. *F. nucleatum* infection at an MOI of 100 induced the highest mRNA expression of IL-8 at 24 h, 48 and 72 h (Figure 3E). The gene expression level of IL-1β was significantly elevated by *F. nucleatum* infection at MOIs of 10, 50, and 100 at 24, 48, and 72 h, and the maximum gene expression level of IL-1β appeared at 48 h infection with *F. nucleatum* at an MOI of 100 (Figure 3F). However, at the protein level, compared with the control group, IL-6 and IL-8 levels in the groups co-cultured with *F. nucleatum* at MOIs of 10, 50, and 100 were significantly elevated; there were no significant differences among the groups infected with different concentrations of *F. nucleatum* (Figures 3G,H). The protein level of IL-1β in the *F. nucleatum*-infected groups was not significantly changed compared with that in the control group (Figure 3I).

### *F. nucleatum* Inhibited the Osteogenic Differentiation of GMSCs by Decreasing the ALP Activity and Mineral Deposition Formation

**Figure 4:**
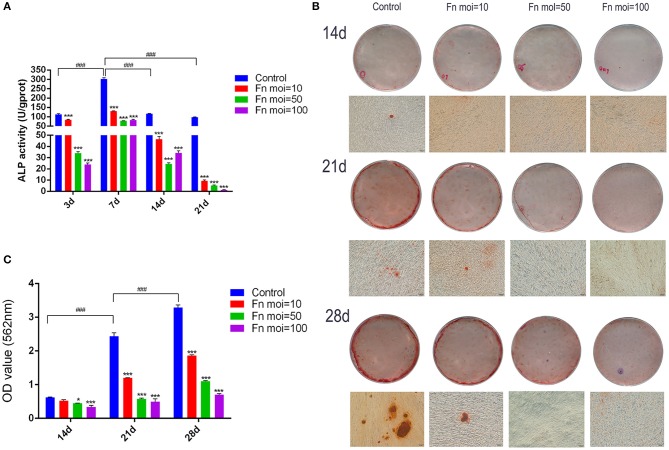
Effects of *F. nucleatum* on GMSC osteogenic differentiation. **(A)** ALP activity in GMSCs co-cultured with *F. nucleatum* (MOIs of 0, 10, 50, and 100) at 3, 7, 14, and 21 d. **(B)** Representative photographs of alizarin red staining of GMSCs at 14, 21, and 28 d with or without *F. nucleatum* infection at MOIs of 10, 50, and 100 by digital camera or microscope; scale bar: 100 μm. **(C)** The concentration of calcium deposited by GMSCs at 14, 21, and 28 d with or without *F. nucleatum* infection at MOIs of 10, 50, and 100. Histograms represent the mean ± SD (*n* = 3). Statistical analyses were performed using two-way ANOVA with Tukey's multiple-comparison test. *P < 0.05 and ***P < 0.001 compared with the control at each time point. ^###^*P* < 0.001.

To explore the effect of *F. nucleatum* infection on GMSCs osteogenic differentiation, the osteogenic differentiation ability in GMSCs co-cultured with time-series *F. nucleatum* were detected by ALP activity assay and alizarin red staining assay. The ALP activity of GMSCs was measured at days 3, 7, 14, and 21 after *F. nucleatum* infection at MOIs of 10, 50, and 100. The results indicated that the ALP activity of uninfected GMSCs peaked on day 7 and *F. nucleatum* dose-dependently decreased the ALP activity in GMSCs co-cultured with *F. nucleatum* at 3, 7, 14, and 21 d (Figure 4A). The mineral deposition in GMSCs with or without *F. nucleatum* infection was measured on days 14, 21, and 28 according to alizarin red staining assay and the relative amount of calcium was quantified by CPC. The results indicated that the mineral deposition of GMSCs increased time-dependently from 14 to 28 d, and large quantities of mineralized nodules were detected at day 28 in the GMSCs without infection by *F. nucleatum*, whereas in GMSCs co-cultured with *F. nucleatum*, the osteogenic medium-induced mineral deposition formation on days 21 and 28 were dose-dependently inhibited by *F. nucleatum* (Figures 4B,C).

### *F. nucleatum* Inhibited the Gene and Protein Expression of Osteogenic Markers in GMSCs

**Figure 5:**
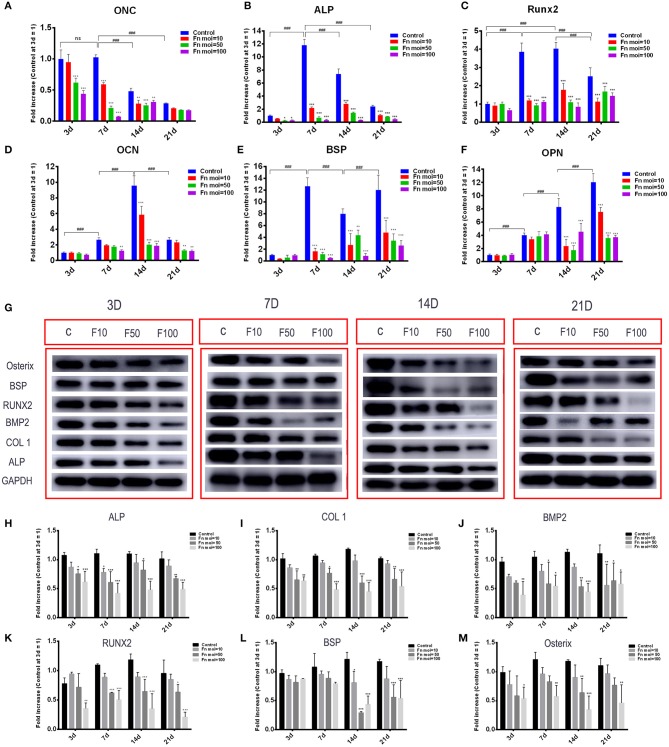
Effects of *F. nucleatum* on the expression of osteogenesis-related genes and proteins in GMSCs. The gene expression of ONC **(A)**, ALP **(B)**, Runx2 **(C)**, OCN **(D)**, BSP **(E)**, and OPN **(F)** in GMSCs co-cultured with *F. nucleatum* (MOIs of 0, 10, 50, and 100) at 3, 7, 14, and 21 d. **(G)** The protein expression of ALP, COL1, BMP2, RUNX2, BSP, osterix on days 3, 7, 14, 21 detected by western blot. The relative protein levels of ALP/GAPDH **(H)**, COL1/GAPDH **(I)**, BMP2/GAPDH **(J)**, RUNX2/GAPDH **(K)**, BSP/GAPDH **(L)**, osterix/GAPDH **(M)**. The histograms represent the mean ± SD (*n* = 3). Statistical analyses were performed using two-way ANOVA with Tukey's multiple-comparison test. ns, no statistic significance. *P < 0.05; **P < 0.01; and ***P < 0.001 compared with the control at each time point. ^###^*P* < 0.001.

To explore the mechanism of *F. nucleatum* inhibited GMSCs osteogenic differentiation ability from the molecular levels, osteogenic related genes and proteins in GMSCs free or co-cultured with *F. nucleatum* were detected. The gene expression levels of *ONC, ALP, Runx2, OCN, BSP*, and *OPN* were detected at 3, 7, 14, and 21 d in GMSCs with or without *F. nucleatum* infection (Figures 5A–F). The results showed that the gene expression levels of *ONC* were increased at day 3 and day 7, and *ALP* expression peaked at day 7. *F. nucleatum* infection at the MOIs of 50 and 100 significantly decreased the gene expression of *ONC* at 3, 7, and 14 d (Figure 5A), and *F. nucleatum* dose-dependently decreased the gene expression of ALP at 7, 14, and 21 d (Figure 5B). For *Runx2*, the gene expression level was increased at day 7; the high expression level was maintained at day 14 and decreased at day 21. *F. nucleatum* infection (MOIs of 10, 50 and 100) significantly downregulated the expression of this gene level at 7, 14, and 21 d (Figure 5C). The expression of OCN, which is a middle-advanced stage marker, peaked at day 14, and *F. nucleatum* infection at an MOI of 100 significantly reduced the gene expression level at 7 d, 14 d and 21 d (Figure 5D). *BSP* is recognized as late osteogenic marker, and the *BSP* gene expression level was significantly decreased by *F. nucleatum* (MOIs of 10, 50 and 100) infection at days 7, 14, and 21 (Figure 5E). For *OPN*, another osteogenic marker, the highest gene expression level appeared on day 21, and *F. nucleatum* infection (MOIs of 10, 50 and 100) dramatically decreased the gene expression level at 14 d and 21 d (Figure 5F). Based on these results, we confirmed *F. nucleatum* at the MOI of 100 significantly inhibited the osteogenic gene expression at various stages. In addition, we confirmed that *F. nucleatum* at an MOI of 100 could dramatically downregulate the expression of the osteogenesis related proteins ALP, COL1, BMP2, Runx2, and Osterix at days 3, 7, 14, and 21. The BSP protein level was decreased by *F. nucleatum* stimulation at days 14 and 21 (Figures 5G–M).

### RNA-seq Analysis of GMSCs Under Time Series *F. nucleatum* Infection

To better understand the global gene expression changes in *F. nucleatum*-infected GMSCs in OM culture conditions over time, we performed a genome-wide transcriptome analysis by RNA-seq. The pearson correlation coefficient analysis and principal coordinates analysis (PCoA) of the genome-wide gene expression levels showed that *F. nucleatum*-infected GMSCs significantly separated from normal cells as the infected time increased (Supplementary Figures 1, 2). In addition, the results indicated that one control sample at 14 d (B14C) and two treated samples at the 14 d (B14F) and 21 d (A21F) time points deviated greatly from the other samples in the same group; thus, these three samples were excluded from the subsequent analysis. These results suggest that the effects of *F. nucleatum* infection on GMSCs may accumulate over time.

**Figure 6:**
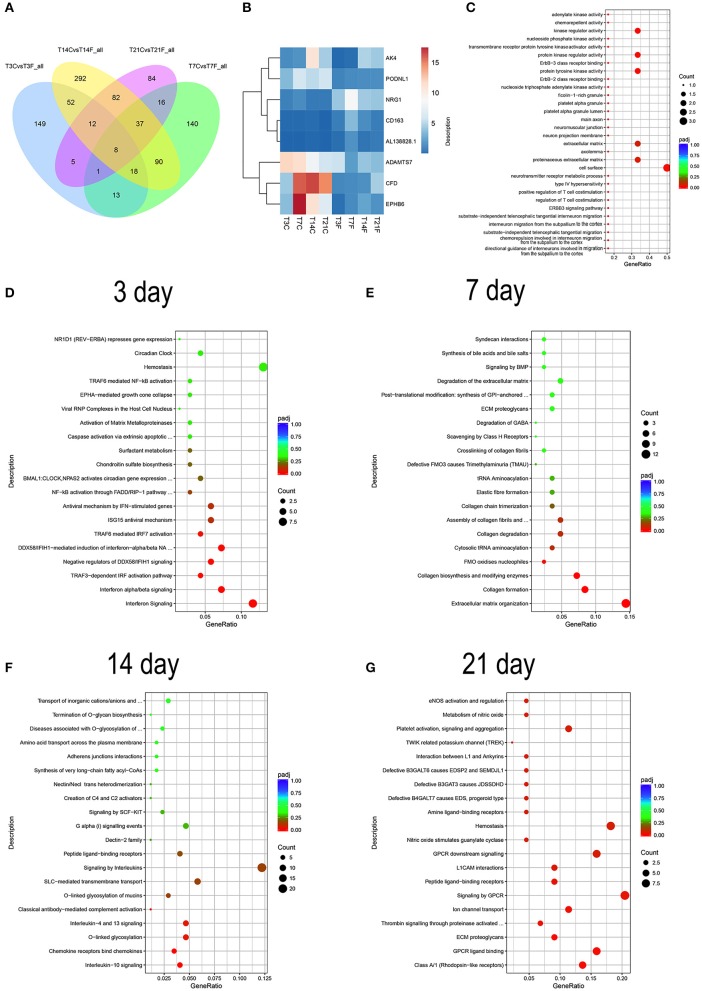
RNA-seq analysis of GMSCs co-cultured with *F. nucleatum* (MOI of 100). GMSCs from 3 different donors were infected by *F. nucleatum* at 3, 7, 14, and 21 d, and the whole gene expression were detected by RNA-seq. T3C, T7C, T14C, and T21C represent the GMSCs in control group without *F. nucleatum* infection at 3, 7, 14, and 21 d; T3F, T7F, T14F, and T21F represent *F. nucleatum* infected GMSCs group at 3, 7, 14, and 21 d, respectively. **(A)** Venn diagram summarizing overlapping DEGs in GMSCs among the four *F. nucleatum* infection time points. T3CvsT3F_all, T7CvsT7F_all, T14CvsT14F_all, and T21CvsT21F_all represent the DEGs generated by *F. nucleatum* infection at 3, 7, 14, and 21 d, respectively. **(B)** The average gene expression level of 8 overlapping DEGs at each group was presented by heatmap. The legend show the gene expression level is increased as the color changes from blue to red. **(C)** The GO enrichment analysis result of the 8 overlapping DEGs. **(D)** DO analysis of the 149 DEGs specifically generated after 3 d of *F. nucleatum* infection. **(E)** DO analysis of the 140 DEGs specifically generated after 7 d of *F. nucleatum* infection. **(F)** DO analysis of the 292 DEGs specifically generated after 14 d of *F. nucleatum* infection. **(G)** DO analysis of the 84 DEGs specifically generated after 21 d of *F. nucleatum* infection. In the legend, the size of the dot represents the number of the DEGs enrichment in the relevant signaling pathway and the colors of the dot represent the Padj value decreased from blue to red **(C–G)**.

To characterize the DEGs affected by *F. nucleatum*, the gene expression profiles of normal GMSCs vs. infected GMSCs at 3, 7, 14, and 21 d were compared at each time point by DEseq2, and 258, 323, 591, 245 DEGs were identified at each time point (Supplementary Figure 3). The overlapping DEGs among the 4 time points included 8 genes that were differentially expressed between normal GMSCs and *F. nucleatum*-infected GMSCs throughout the whole process. In addition, 149, 140, 292, and 84 DEGs were specifically identified at 3, 7, 14, and 21 d of *F. nucleatum* infection, respectively (Figure 6A). The heatmap analysis showed that among the 8 DEGs, the gene expression levels of AK4, PODNL1, ADAMTS7, CFD, and EPHB6 were down-regulated, and those of NRG1, CD163, AL1 were up-regulated (Figure 6B). The GO analysis of the 8 genes indicated that they were involved in kinase regulatory activity, and the extracellular matrix, and enriched in cell migration-related signaling pathways, such as directional guidance of interneurons involved in migration from the subpallium to the cortex (Figure 6C). In addition, the reactome functional enrichment analysis showed that 149 DEGs specifically induced by *F. nucleatum* infection at 3 d were predominantly enriched in the interferon-related signaling pathway (Figure 6D); 140 DEGs specifically produced after 7 d of *F. nucleatum* infection were mostly enriched in extracellular matrix organization, collagen formation, collagen biosynthesis, and modifying enzymes (Figure 6E), and 292 unique DEGs generated after 14 d of *F. nucleatum* infection were mainly enriched in IL-10, IL-4, and IL-13 signaling, chemokine receptors bind chemokines, and O-linked glycosylation signaling pathway (Figure 6F); finally, 84 DEGs specifically produced at 21 d of *F. nucleatum* infection were largely enriched in class A/1 (rhodopsin-like receptors) and GPCR-related signaling pathway (Figure 6G). Furthermore, we identified a total of 999 DEGs that were generated after *F. nucleatum* infection at 4 time points, and all these DEGs could be divided into 4 clusters (Supplementary Figure 4 and Supplementary Table 4).

**Figure 7:**
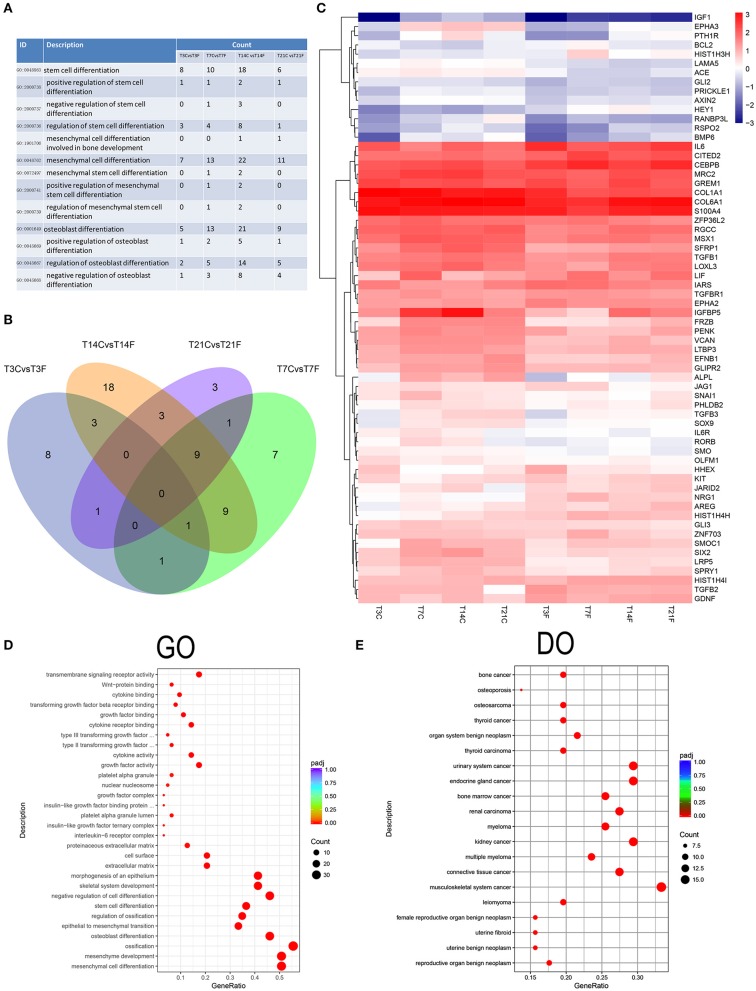
Analysis of the cell differentiation-related DEGs. The GMSCs from 3 different donors were infected by *F. nucleatum* at 3, 7, 14, and 21 d, and the whole gene expression were detected by RNA-seq. The osteogenic differentiation related DEGs was identified and further analyzed. **(A)** The cell differentiation-related biological process GO terms were identified and the DEGs number in GMSCs after *F. nucleatum* infection at 3, 7, 14, and 21 d were calculated compared with control group at each time point. **(B)** The cell differentiation-related DEGs generated after *F. nucleatum* infection at 3, 7, 14, and 21 d were overlapped by Venn diagram. T3CvsT3F, T7CvsT7F, T14CvsT14F, and T21CvsT21F represent the osteogenic differentiation related DEGs numbers generated by *F. nucleatum* infection at 3, 7, 14, and 21 d, respectively. **(C)** The average genes expression level of the 64 union cell differentiation related DEGs generated by *F. nucleatum* infection at 4 time points at each group were presented by heatmap. The legend show the gene expression level is increasing as the color changes from blue to red. The GO **(D)** and DO **(E)** enrichment analysis of the 64 unique cell differentiation-related DEGs. In the legend, the size of the dot represents the number of the DEGs enrichment in the relevant signaling pathway and the colors of the dot represent the Padj value decreased from blue to red.

To further investigate the effect of *F. nucleatum* on GMSC differentiation, the expression signatures of stem cell differentiation-related DEGs involved in the GO biological processes were selected (Figure 7A). The Venn diagram revealed a total of 64 DEGs; the DEGs varied with increasing *F. nucleatum* infection time, and no overlapping DEGs were generated (Figure 7B), which suggests that GMSC osteogenic differentiation is regulated by various genes at different stages during *F. nucleatum* infection. The heatmap comprehensively showed the gene expression levels of 64 genes, including Bcl2, IL-6, and TGFb2, which indicates inflammation and apoptosis related genes were activated by *F. nucleatum* during the process of GMSCs osteogenic differentiation (Figure 7C). The GO function analysis confirmed the following: (1) at the molecular function level, all of the DEGs were enriched in cytokine activity, cytokines and growth factor binding-related signaling pathway; (2) at the cellular component level, all of the DEGs were enriched in growth factor complex, cell surface, and extracellular matrix signaling pathways; and (3) at the biological process level, all of the DEGs were enriched in stem cell differentiation, and mesenchymal cell differentiation signaling pathways, which indicates that the biological processes of GMSC development and differentiation are closely related to cytokine production and related receptor activation in the *F. nucleatum* induced microenvironment (Figure 7D). Interestingly, we discovered that 64 cell differentiation-related DEGs were associated with human cancers, such as bone cancer, osteosarcoma, and thyroid cancer according to the DO enrichment analysis (Figure 7E). The KEGG and DisGenet analyses also confirmed that the DEGs were significantly enriched in cancer-related pathways, such as colorectal cancer and fibroid tumor, and so on (Supplementary Figure 5), which suggests that persistent exposure to *F. nucleatum* greatly increases the risk of GMSC carcinogenesis during the process of osteogenic differentiation. The protein-protein interaction network of 64 DEGs showed a potentially complex interaction relationship among these genes (Supplementary Figure 6).

## Discussion

Polymicrobial infection with *Porphyromonas gingivalis* and *F. nucleatum* has been shown to can aggravate periodontal alveolar bone loss and induce an increased inflammatory response in a mouse periodontitis model (Polak et al., 2009). The stimulatory mechanism of inflammatory bone resorption in periodontitis induced by *P. gingivalis* has been comprehensive reported (Hanazawa, 1998; Jia et al., 2019), whereas the defined effect of *F. nucleatum* on oral tissue destruction has rarely been explored. Our current study confirms that *F. nucleatum* significantly alters the biological properties of GMSCs by promoting cell migration and chemokine/cytokine release and decreasing the proliferation and osteogenic differentiation ability. The large number of inflammatory cytokines release and the decreased osteogenic differentiation ability of GMSCs are critical in periodontal supporting tissue destruction.

In this study, we demonstrate that *F. nucleatum* inhibits the proliferation of GMSCs. It has been reported that *F. nucleatum* can impair endothelial cell and mononuclear cell proliferation, which is consistent with our finding in GMSCs (Haake and Lindemann, 1997; Mendes et al., 2016). Viable and formaldehyde-treated, but not sonicated or heat-treated *F. nucleatum* bacteria, can cause severe aggregation and apoptosis of peripheral blood mononuclear cells (PBMCs) (Huynh et al., 2011). Bartold et al. reported that *F. nucleatum* inhibits human gingival fibroblast proliferation by releasing ammonium and butyrate (Bartold et al., 1991). However, *F. nucleatum* promotes colorectal cancer cell (CRC) line proliferation by modulating the E-cadherin/β-catenin signaling pathway or activating Toll-like receptor 4 signaling to nuclear factor-kB (Rubinstein et al., 2013; Yang et al., 2017). *F. nucleatum* could promote CRC line proliferation but inhibit the GMSCs proliferation, which suggested the mechanism of *F. nucleatum* regulated cell proliferation is different in cancer cell lines and primary somatic cells. In the future, we would explore the mechanism of *F. nucleatum* on GMSCs proliferation inhibition by separating the detailed bacterial components and evaluating the effect of each component on GMSCs proliferation.

In human leukemic monocyte cells, *F. nucleatum* can trigger an inflammatory response by upregulating the expression of tumor necrosis factor-α, IL-1β, IL-6, IL-8, and monocyte chemoattractant protein-1 and promote the recruitment and transmigration of monocytes through endothelial cells (Wang et al., 2019). Uitto et al. reported that *F. nucleatum* increase infected epithelial cell migration and survival by increasing collagenase 3 production (Uitto et al., 2005). *F. nucleatum* infection leads to the recruitment of macrophages and osteoclasts, finally resulting in gingival inflammation and bone resorption (Johnson et al., 2018). In human GECs, *F. nucleatum* upregulates the gene expression levels of IL-1, IL-6, IL-8, SOD2, CCL20, CXCL1, CXCL3, and CSF2 partly by activating the NF-κB, mitogen-activated protein kinase (MAPK) p38, and MAPK kinase/extracellular signal-regulated kinase (MEK/ERK) pathways (Huang et al., 2004; Milward et al., 2007; Ramage et al., 2017). *F. nucleatum* increases the production of PTGS2, PGE2, and the ratio of RANKL/OPG in periodontal ligament (PDL) cells, which play key roles in the inflammation and destruction in periodontitis (Nogueira et al., 2014). Bui et al. reported that *F. nucleatum* could invasive into GECs and then leads to the activation of NLRP3 inflammasome, which finally resulted in the secretion of IL-1β and the elevated protein level could be detected, which was different with our findings in *F. nucleatum* infected GMSCs (Bui et al., 2016). In the current study, we preliminarily confirmed that *F. nucleatum* could promote GMSC migration, which is consist with the results of *F. nucleatum* on epithelial cell, monocytes, macrophages and osteoclasts. In addition, we confirmed *F. nucleatum* could increase the chemokines and inflammatory cytokines releases, such as CCL2, CXCL1, PTGS2, IL-6, and IL-8, which is consistent with the previous studies on *F. nucleatum* infection with GECs and PDL cells. However, the IL-1β expression in *F. nucleatum* infected GMSCs is inconsistent with GECs, which indicated that the mechanism of *F. nucleatum* on GMSCs and GECs are different. Our current study is limited for lack of the detailed molecular mechanism by which *F. nucleatum* promotes GMSC migration and inflammatory cytokine production.

The periodontal supporting tissues regeneration based on stem cells has been regarded as a novel therapeutic strategy to repair the periodontal defects (Iohara et al., 2004; Liu et al., 2008). Tissue regeneration is an organized dynamic process, including hemostasis, an inflammatory phase, proliferation, and maturation/matrix remodeling; external factors and the infection by pathogenic microorganisms could largely impact the whole process of periodontal tissues regeneration (Hammerle and Giannobile, 2014). GMSCs have attracted a great deal of attention in periodontal regenerative therapy research because of their ease of accessibility, well-established self-renewal, multipotent differentiation, and immune modulatory properties (Hammerle and Giannobile, 2014; Diniz et al., 2016; Fawzy and Dorfer, 2016). However, current study on stem cell research is defective for few concerned on the osteogenic differentiation ability of stem cells in the inflammatory microenvironment. In our study, we firstly confirmed that *F. nucleatum* significantly inhibited GMSC osteogenic differentiation by decreasing the osteogenesis-related gene and protein expression, ALP activity and mineralized nodule formation. Our transcriptome analysis results showed that *F. nucleatum* disturbed GMSC gene expression during osteogenic differentiation, and some DEGs are significantly enriched in cancer-related signaling pathways, which suggested that persistent *F. nucleatum* infection had the potential to induce GMSCs progression into cancer cells. Previous studies indicated that *F. nucleatum* was closely associated with various types of cancer, such as colorectal cancer, gastric cancer, and pancreatic cancer (Amitay et al., 2017; Hsieh et al., 2018), which suggested that it was worth to focus on tumorigenesis during the process of long-term stem cell-based regeneration therapy.

In summary, we confirmed that *F. nucleatum* played multi-dimensional roles in altering the biological properties of GMSCs, including inhibiting GMSC proliferation, promoting cell migration and chemokine/cytokine generation, such as CCL2, CCL20, CXCL1, PTGS2, IL-6, IL-8, and IL-1β. Moreover, *F. nucleatum* decreases GMSC osteogenic differentiation partly by decreasing ALP activity and mineralized nodule formation, and downregulating osteogenesis-related gene and protein expression. Additionally, a time course RNA-seq analysis indicated that *F. nucleatum* activated cellular signaling pathways in a time-dependent manner. In addition, we identified a total of 64 cell differentiation-related DEGs in GMSCs with or without *F. nucleatum* infection at 3, 7, 14, and 21 d. Intriguingly, we discovered that the cell differentiation-related DEGs generated in *F. nucleatum*-infected GMSCs are significantly enriched in cancer-related pathways, such as bone cancer, osteosarcoma and bone marrow cancer. Our study is the first to illuminate the multidimensional biological effects of *F. nucleatum* on GMSCs and makes a foundation for future mechanistic studies of GMSCs under *F. nucleatum* infection. However, our current study is limited due to the lack of detailed molecular experiments to evaluate the mechanism by which *F. nucleatum* inhibits GMSC proliferation and osteogenic differentiation and promotes migration and cytokine generation. In the future, we will construct a large number of *F. nucleatum* mutant strains, establish *in vivo* models, and perform a series of molecular biology experiments, such as skin injury models of migration and cell immunofluorescence assays to explore the comprehensive effects and mechanisms by which *F. nucleatum* affects GMSC proliferation, migration, and osteogenic differentiation.

## Data Availability Statement

The datasets generated for this study can be found in the NCBIs Gene Expression Omnibus (GEO, http://www.ncbi.nlm.nih.gov/geo/) and are accessible through GEO series accession number (GSE126821).

## Ethics Statement

This study was approved by the Medical Ethical Committee of Stomatology School, Shandong University (Protocol Number: 20170101). All individuals were informed with this research project and signed the informed consent according to the Helsinki Declaration of 1975.

## Author Contributions

QF conceived, designed, and supervised this study. WK and DT collected samples. WK performed experiments and analyzed the data. WK and QF wrote the paper. XJ, XZ, WK, and QF revised the manuscript.

### Conflict of Interest

The authors declare that the research was conducted in the absence of any commercial or financial relationships that could be construed as a potential conflict of interest.

## Supplementary Material

Supplementary Figure 1 RNA-seq analysis of GMSCs co-cultured with *F. nucleatum* (MOI of 100). GMSCs from 3 different donors were infected by *F. nucleatum* at 3, 7, 14, and 21 d. The correlation of the 24 samples was showed according to the pearson correlation coefficient analysis. The first letter in the sample name represents the donor ID, the middle number represents the time, and the last number C and F represent control and *F. nucleatum*, respectively. For example, A3C represent the sample from donor A that growth for 3 days without *F. nucleatum* infection; A3F represent the sample from donor A that growth for 3 d with *F. nucleatum* infection.

Supplementary Figure 2 RNA-seq analysis of GMSCs co-cultured with *F. nucleatum* (MOI of 100). GMSCs from 3 different donors were infected by *F. nucleatum* at 3, 7, 14, and 21 d. The principal coordinates analysis (PCoA) of the genome-wide gene expression levels showed *F. nucleatum*-infected GMSCs significantly separated from normal cells as the infected time increased. The first letter in the sample name represents the donor ID, the middle number represents the time, and the last number C and F represent control and *F. nucleatum*, respectively. In the legend, T3C, T7C, T14C, and T21C represent control group at 3, 7, 14, and 21 d respectively. T3F, T7F, T14F, and T21F represent *F. nucleatum*-infected group at 3, 7, 14, and 21 d, respectively. For example, T3C represents the union of A3C, B3C, and C3C; T3F represents the union of A3F, B3F, and C3F.

Supplementary Figure 3 RNA-seq analysis of GMSCs co-cultured with *F. nucleatum* (MOI of 100). GMSCs from 3 different donors were infected by *F. nucleatum* at 3, 7, 14, and 21 d. The number of DEGs after *F. nucleatum* infection at 3, 7, 14, and 21 d were calculated. The red dot represents the up-regulated genes; the green dot represents the down-regulated genes; and the blue dot represents the genes not influenced by *F. nucleatum* infection. The gene numbers regulated by *F. nucleatum* are showed in the legend.

Supplementary Figure 4 The GMSCs from 3 different donors were infected by *F. nucleatum* at 3, 7, 14, and 21 d, and the whole gene expression were detected by RNA-seq. Compared with control group at each time point, the DEGs generated in GMSCs after *F. nucleatum* infection at 3, 7, 14, and 21 d were identified. The average genes expression level of the 999 union DEGs generated by *F. nucleatum* infection at 4 time points at each group were presented by heatmap (absolute of the fold-change of DEGs > 2 and an adjusted *P* < 0.01). T3C, T7C, T14C, and T21C represent control group at 3, 7, 14, and 21 d, respectively. T3F, T7F, T14F, and T21F represent *F. nucleatum* infected group at 3, 7, 14, and 21 d, respectively. For example, T3C represents the union of A3C, B3C, and C3C; T3F represents the union of A3F, B3F, and C3F. The legend show the gene expression level is increasing as the color changes from blue to red.

Supplementary Figure 5 The GMSCs from 3 different donors were infected by *F. nucleatum* at 3, 7, 14, and 21 d, and the whole gene expression were detected by RNA-seq. The 64 unique osteogenic differentiation-related DEGs were enriched in signaling pathways according to KEGG **(A)** and DisGeNET **(B)** database. In the legend, the size of the dot represents the number of the DEGs enrichment in the relevant signaling pathway and the colors of the dot represent the Padj value decreased from blue to red.

Supplementary Figure 6 The GMSCs from 3 different donors were infected by *F. nucleatum* at 3, 7, 14, and 21 d, and the whole gene expression were detected by RNA-seq. A total of 64 unique osteogenic differentiation-related DEGs were identified after *F. nucleatum* infection at 3, 7, 14, 21 d, and the complex interactive network of these 64 DEGs were visualized based on the STRING database. All the genes are presented by the color nodes, and the node content represents the 3D structure of the proteins. The known, predicated or other protein-protein interaction associations are presented by different color edges.

Supplementary Table 1 Primers sequences for quantitative PCR of *F. nucleatum*.

Supplementary Table 2 Primers sequences for quantitative real-time PCR of GMSCs.

Supplementary Table 3 Primary antibodies used in the assay of western blot.

Supplementary Table 4 The union 999 DEGs list of GMSCs infected by *F. nucleatum* at day 3, 7, 14, and 21 (absolute of the fold-change of DEGs >2 and an adjusted *P* < 0.01).
