# Supplementary figures and images for: Persistent Exposure to Fusobacterium nucleatum Triggers Chemokine/Cytokine Release and Inhibits the Proliferation and Osteogenic Differentiation Capabilities of Human Gingiva-Derived Mesenchymal Stem Cells
Source: Front Cell Infect Microbiol. 2019 Dec 17;9:429. doi: 10.3389/fcimb.2019.00429 (PMC6927917; doi:10.3389/fcimb.2019.00429)

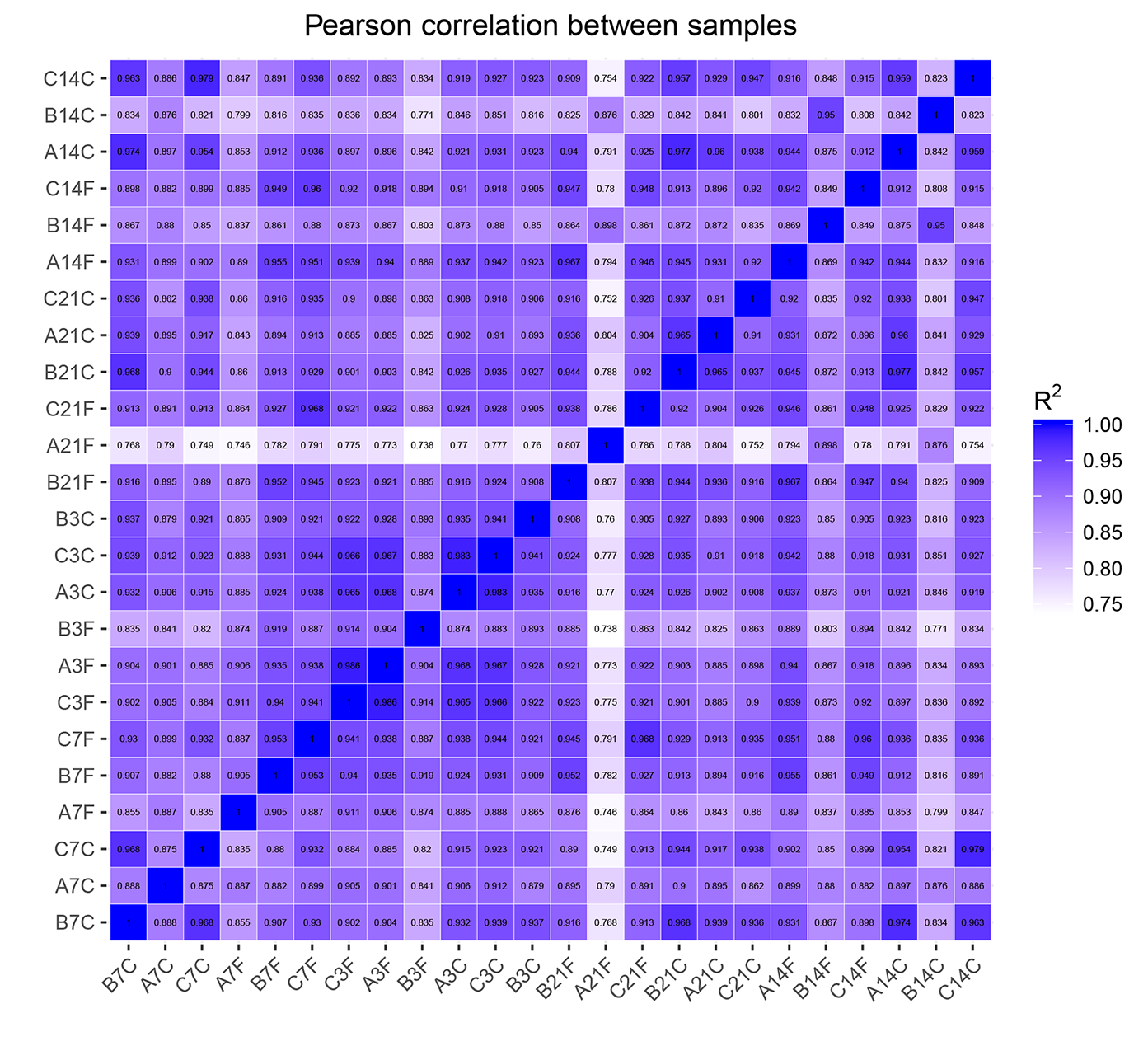

Supplement: Supplementary Figure 1 — RNA-seq analysis of GMSCs co-cultured with F. nucleatum (MOI of 100). GMSCs from 3 different donors were infected by F. nucleatum at 3, 7, 14, and 21 d. The correlation of the 24 samples was showed according to the pearson correlation coefficient analysis. The first letter in the sample name represents the donor ID, the middle number represents the time, and the last number C and F represent control and F. nucleatum, respectively. For example, A3C represent the sample from donor A that growth for 3 days without F. nucleatum infection; A3F represent the sample from donor A that growth for 3 d with F. nucleatum infection. [file Image_1.TIF]

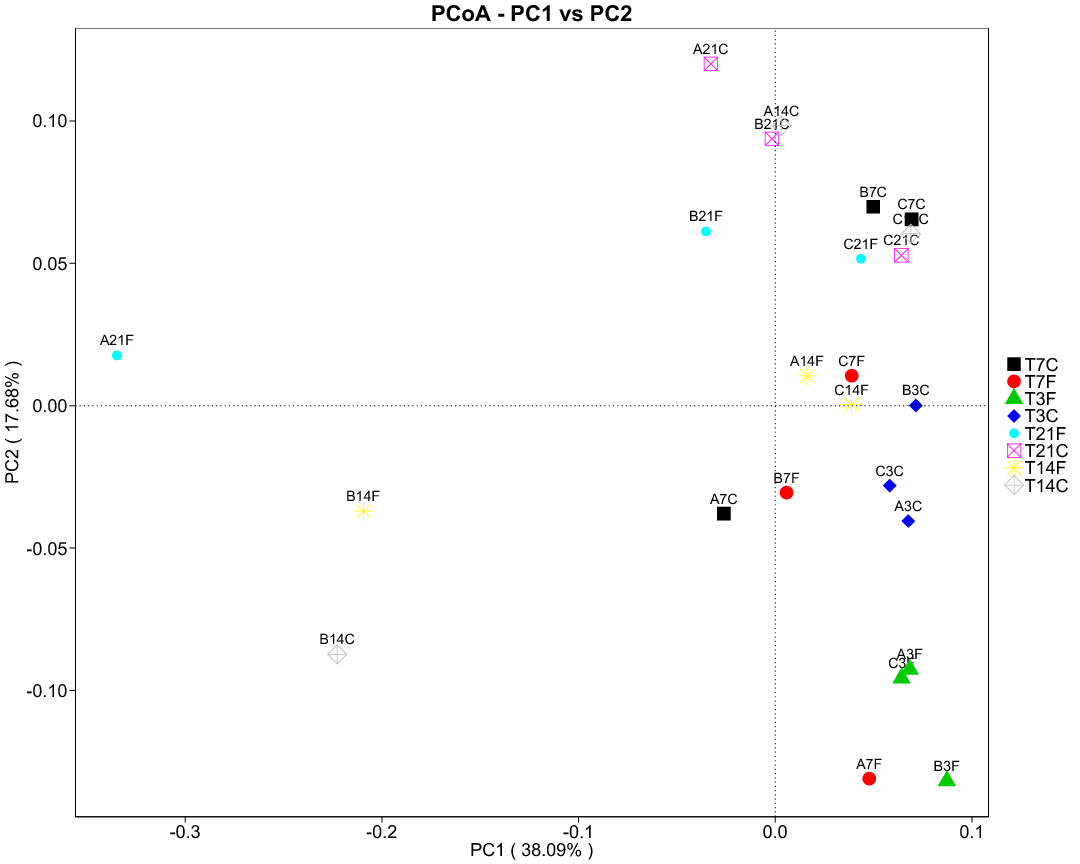

Supplement: Supplementary Figure 2 — RNA-seq analysis of GMSCs co-cultured with F. nucleatum (MOI of 100). GMSCs from 3 different donors were infected by F. nucleatum at 3, 7, 14, and 21 d. The principal coordinates analysis (PCoA) of the genome-wide gene expression levels showed F. nucleatum-infected GMSCs significantly separated from normal cells as the infected time increased. The first letter in the sample name represents the donor ID, the middle number represents the time, and the last number C and F represent control and F. nucleatum, respectively. In the legend, T3C, T7C, T14C, and T21C represent control group at 3, 7, 14, and 21 d respectively. T3F, T7F, T14F, and T21F represent F. nucleatum-infected group at 3, 7, 14, and 21 d, respectively. For example, T3C represents the union of A3C, B3C, and C3C; T3F represents the union of A3F, B3F, and C3F. [file Image_2.TIF]

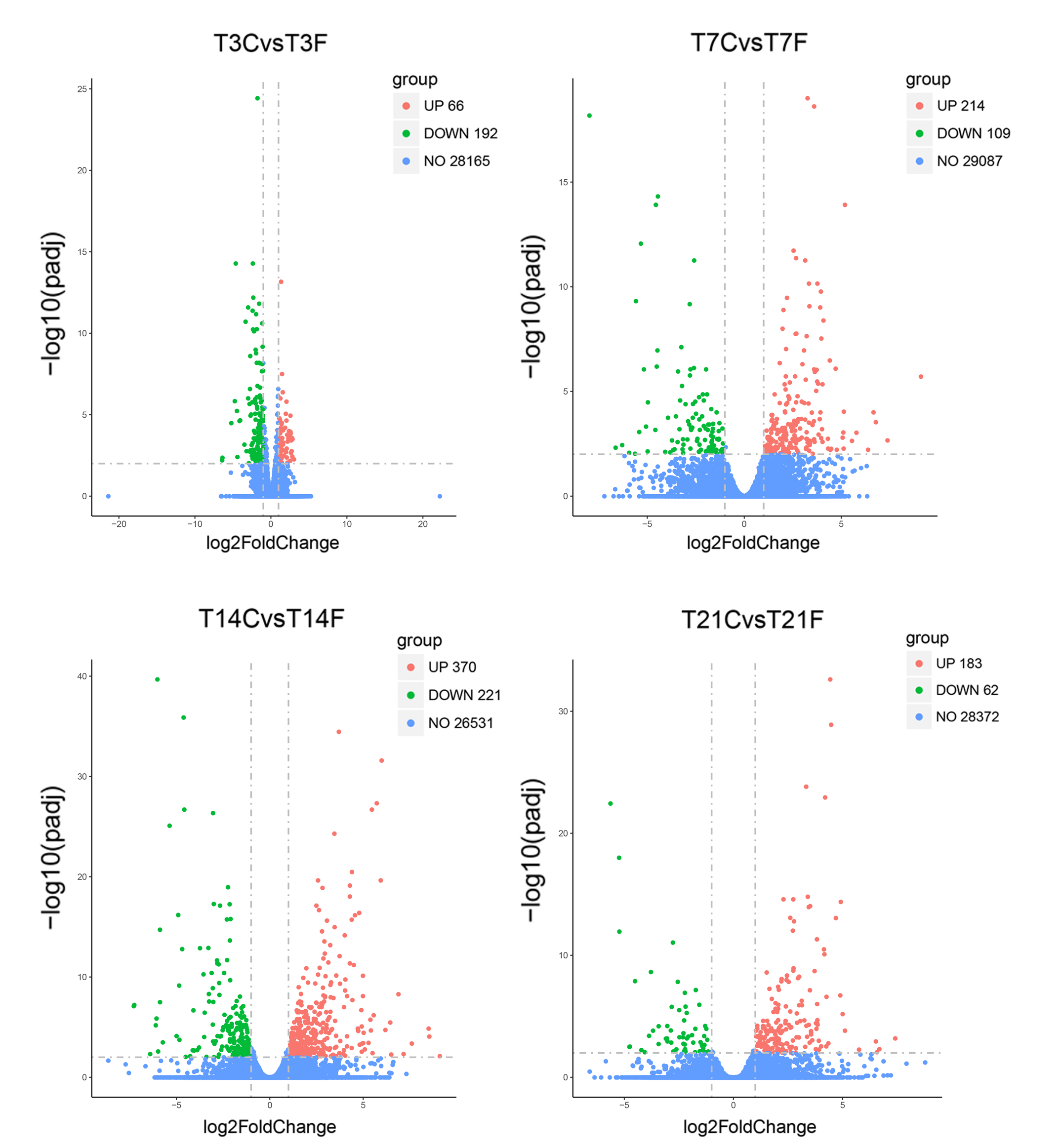

Supplement: Supplementary Figure 3 — RNA-seq analysis of GMSCs co-cultured with F. nucleatum (MOI of 100). GMSCs from 3 different donors were infected by F. nucleatum at 3, 7, 14, and 21 d. The number of DEGs after F. nucleatum infection at 3, 7, 14, and 21 d were calculated. The red dot represents the up-regulated genes; the green dot represents the down-regulated genes; and the blue dot represents the genes not influenced by F. nucleatum infection. The gene numbers regulated by F. nucleatum are showed in the legend. [file Image_3.TIF]

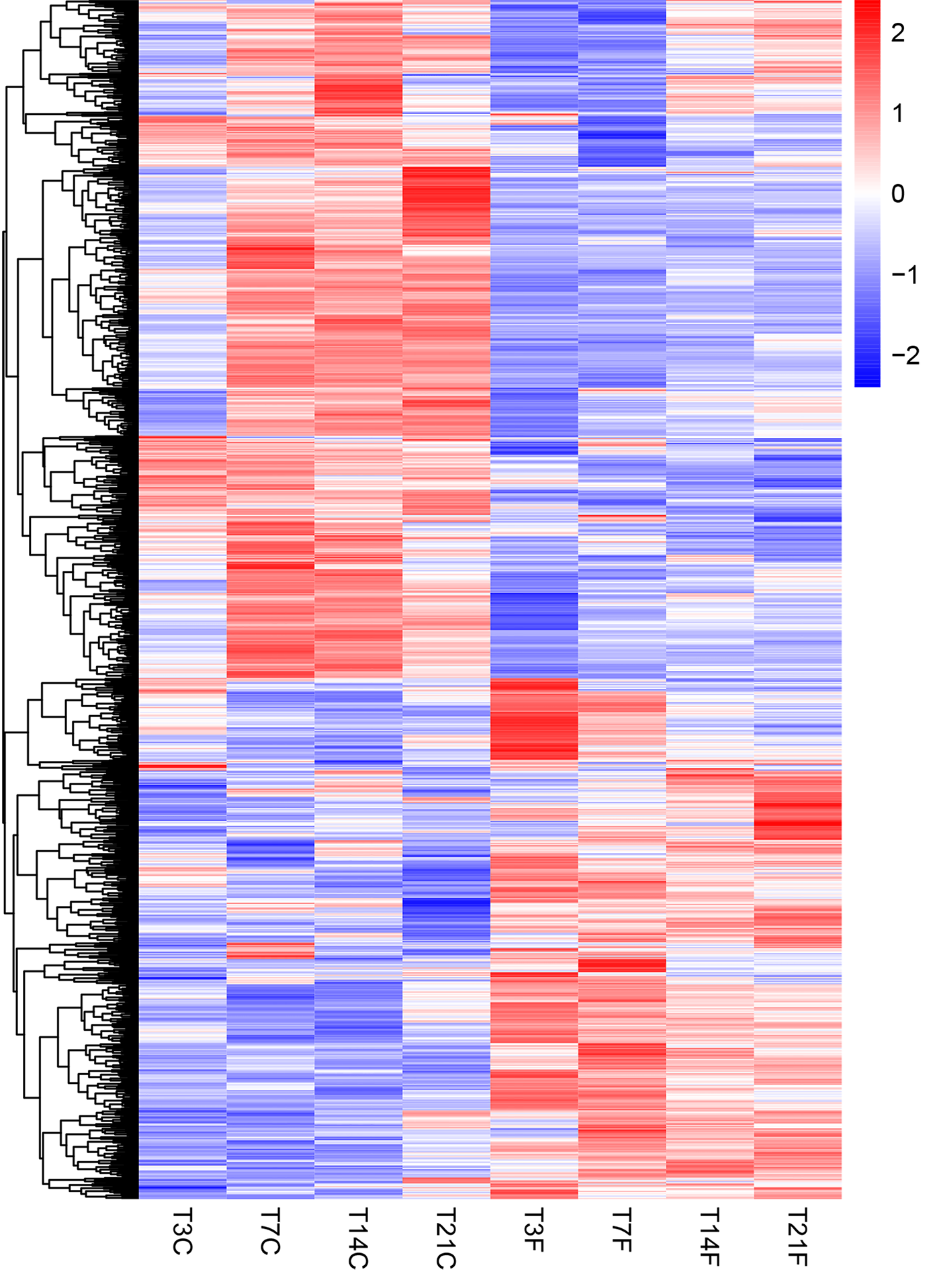

Supplement: Supplementary Figure 4 — The GMSCs from 3 different donors were infected by F. nucleatum at 3, 7, 14, and 21 d, and the whole gene expression were detected by RNA-seq. Compared with control group at each time point, the DEGs generated in GMSCs after F. nucleatum infection at 3, 7, 14, and 21 d were identified. The average genes expression level of the 999 union DEGs generated by F. nucleatum infection at 4 time points at each group were presented by heatmap (absolute of the fold-change of DEGs > 2 and an adjusted P < 0.01). T3C, T7C, T14C, and T21C represent control group at 3, 7, 14, and 21 d, respectively. T3F, T7F, T14F, and T21F represent F. nucleatum infected group at 3, 7, 14, and 21 d, respectively. For example, T3C represents the union of A3C, B3C, and C3C; T3F represents the union of A3F, B3F, and C3F. The legend show the gene expression level is increasing as the color changes from blue to red. [file Image_4.TIF]

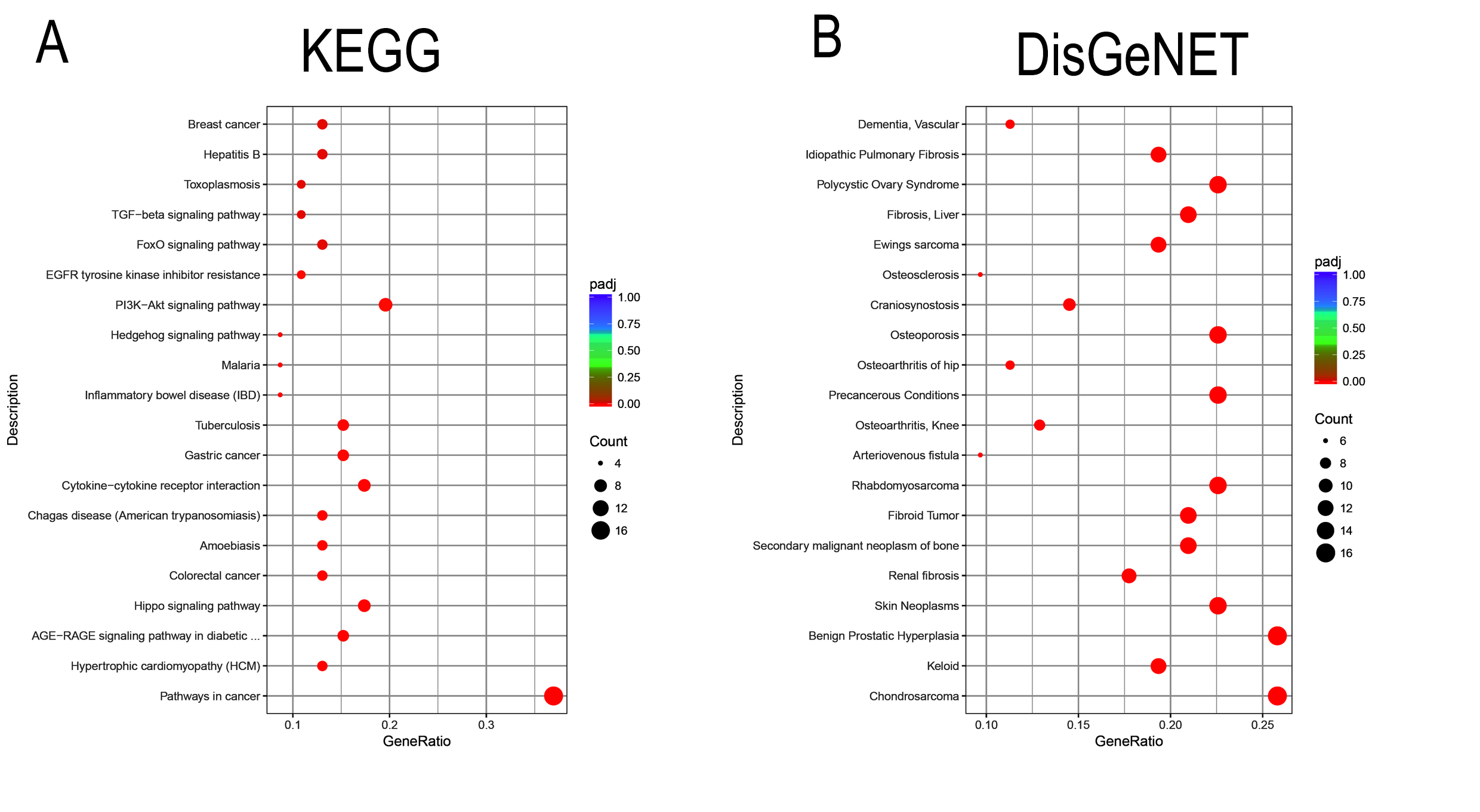

Supplement: Supplementary Figure 5 — The GMSCs from 3 different donors were infected by F. nucleatum at 3, 7, 14, and 21 d, and the whole gene expression were detected by RNA-seq. The 64 unique osteogenic differentiation-related DEGs were enriched in signaling pathways according to KEGG (A) and DisGeNET (B) database. In the legend, the size of the dot represents the number of the DEGs enrichment in the relevant signaling pathway and the colors of the dot represent the Padj value decreased from blue to red. [file Image_5.TIF]

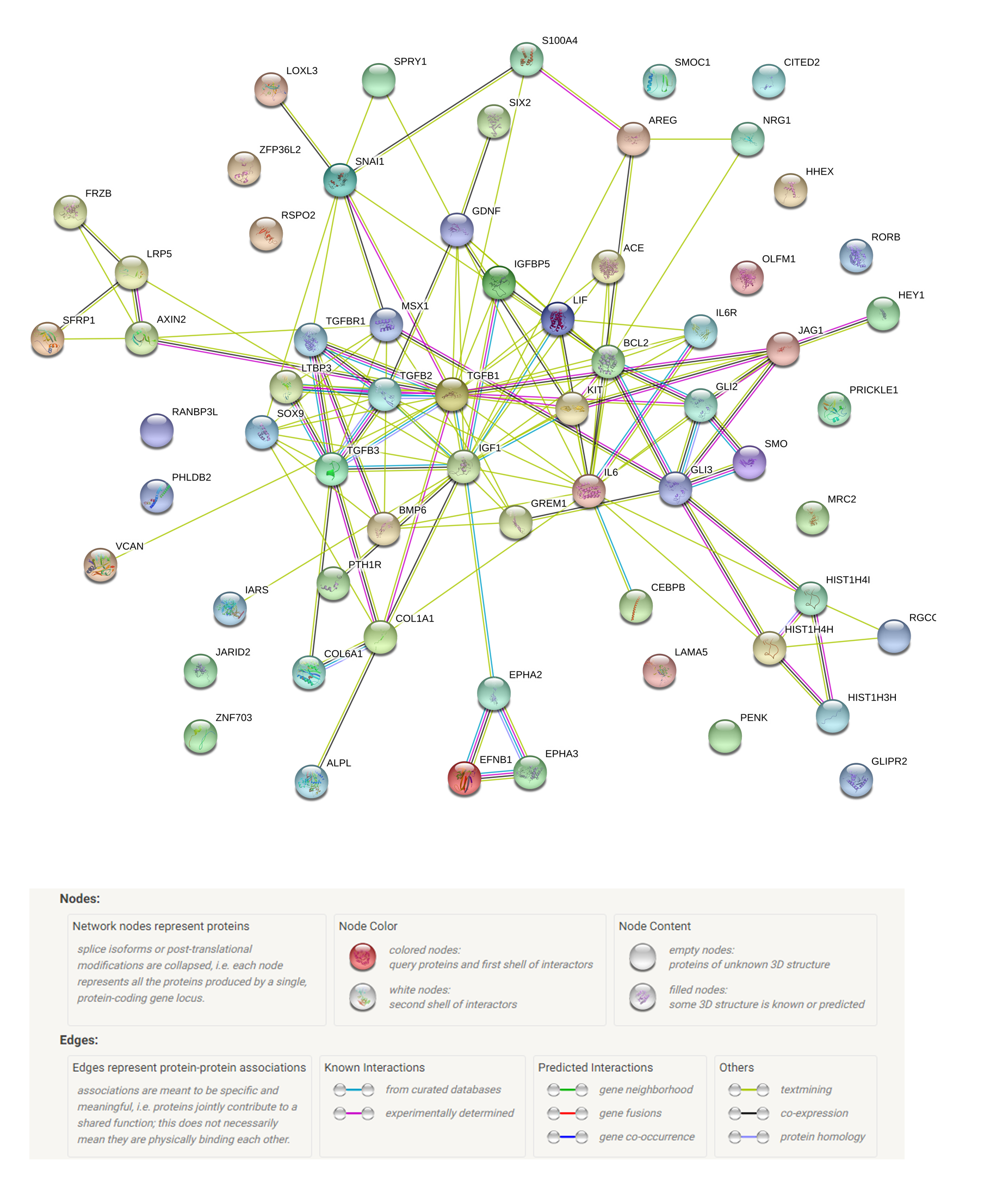

Supplement: Supplementary Figure 6 — The GMSCs from 3 different donors were infected by F. nucleatum at 3, 7, 14, and 21 d, and the whole gene expression were detected by RNA-seq. A total of 64 unique osteogenic differentiation-related DEGs were identified after F. nucleatum infection at 3, 7, 14, 21 d, and the complex interactive network of these 64 DEGs were visualized based on the STRING database. All the genes are presented by the color nodes, and the node content represents the 3D structure of the proteins. The known, predicated or other protein-protein interaction associations are presented by different color edges. [file Image_6.TIF]
