# Supplementary material for: Persistent Exposure to Fusobacterium nucleatum Triggers Chemokine/Cytokine Release and Inhibits the Proliferation and Osteogenic Differentiation Capabilities of Human Gingiva-Derived Mesenchymal Stem Cells
Source: Front Cell Infect Microbiol. 2019 Dec 17;9:429. doi: 10.3389/fcimb.2019.00429 (PMC6927917; doi:10.3389/fcimb.2019.00429)
Supplement: Supplementary Table 1 — Primers sequences for quantitative PCR of F. nucleatum. [file Table_1.docx]

**Table S1: Primers sequences for qPCR measurement of** *F. nucleatum*

| Gene | Primer sequences | | |
| --- | --- | --- | --- |
|  | 5ˊ~3ˊForward | 5ˊ~3ˊReverse | |
| 16S  *F. nucleatum* | GAGTTTGATCATGGCTCAG  TTGTAAGTGCTGGTAAAGGGATTG | | AAGGAGGTGATCCAGCCGCA  CATTCCTACATAACGTCAAGAGGTA |
