# Supplementary material for: Persistent Exposure to Fusobacterium nucleatum Triggers Chemokine/Cytokine Release and Inhibits the Proliferation and Osteogenic Differentiation Capabilities of Human Gingiva-Derived Mesenchymal Stem Cells
Source: Front Cell Infect Microbiol. 2019 Dec 17;9:429. doi: 10.3389/fcimb.2019.00429 (PMC6927917; doi:10.3389/fcimb.2019.00429)
Supplement: Supplementary Table 2 — Primers sequences for quantitative real-time PCR of GMSCs. [file Table_2.DOCX]

**Table S2. Primers sequences for quantitative real-time PCR (qRT-PCR)**

| Gene | Primer sequences | | |
| --- | --- | --- | --- |
|  | 5ˊ~3ˊForward | 5ˊ~3ˊReverse | |
| GAPDH  CCL2  CCL5  CCL20  CXCL1  CXCL2  CXCL3  CSF2  PTGS2  SOD2  IL-6  IL-8  IL-1β  ONC  ALP  Runx2  OPN  BSP  OCN | GCACCGTCAAGGCTGAGAAC  CAGCCAGATGCAATCAATGCC  TGCTGCTTTGCCTACATTGC  CGGCGAATCAGAAGCAAGC  ATCGAAAAGATGCTGAACAGTGAC  CTTGTCTCAACCCCGCATCG  GCCCAAACCGAAGTCATAGC  ACTTCCTGTGCAACCCAGATT  GCTGTTCCCACCCATGTCAA  GGGATTGATGTGTGGGAGCA  ATAACCACCCCTGACCCAAC  TCAGAGACAGCAGAGCACAC  CTTTGAAGCTGATGGCCCTAA  GGTGGTGGAAGAAACTGTGG  ATGGGATGGGTGTCTCCACA  TCCACACCATTAGGGACCATC  TCCTAGCCCCACAGACCCTT  CCCCACCTTTTGGGAAAACCA  TCACACTCCTCGCCCTATT | | TGGTGAAGACGCCAGTGGA  TGGAATCCTGAACCCACTTCT  CATCCTTGACCTGTGGACGA  TTGGATTTGCGCACACAGAC  TTCAGGAACAGCCACCAGTGA  AGTTGGATTTGCCATTTTTCAGC  TGGTGCTCCCCTTGTTCAGT  TCATCTGGCCGGTCTCACTC  AAATTCCGGTGTTGAGCAGT  CATAAAGAGCTTAACATACTCAGCA  CCCATGCTACATTTGCCGAA  GGCAAAACTGCACCTTCACA  AGTGGTGGTCGGAGATTCGT  AGAAGTGGCAGGAAGACTCG  CCACGAAGGGGAACTTGTC TGCTAATGCTTCGTGTTTCCA  CACACTATCACCTCGGCCAT  TCCCCGTTCTCACTTTCATAGAT  GATGTGGTCAGCCAACTCG-3 |

GAPDH: glyceraldehyde-3-phosphate dehydrogenase; CXC: C-X-C motif chemokine ligand; CSF: colony stimulating factor; PTGS2: prostaglandin-endoperoxide synthase 2; SOD2: superoxide dismutase 2; ALP: alkaline phosphatase; Runx2: runt related transcription factor 2; OPN: osteopontin; BSP: bone sialoprotein; OCN: osteocalcin; ONC: Osteonectin.
