# Supplementary material for: Persistent Exposure to Fusobacterium nucleatum Triggers Chemokine/Cytokine Release and Inhibits the Proliferation and Osteogenic Differentiation Capabilities of Human Gingiva-Derived Mesenchymal Stem Cells
Source: Front Cell Infect Microbiol. 2019 Dec 17;9:429. doi: 10.3389/fcimb.2019.00429 (PMC6927917; doi:10.3389/fcimb.2019.00429)
Supplement: Supplementary Table 3 — Primary antibodies used in the assay of western blot. [file Table_3.DOCX]

**Table S3. Primary antibodies for western blot**

| Antibody  (Catalog number) | Company | dilution | Molecular Weight (kDa) |
| --- | --- | --- | --- |
| Osterix (ab209484) | Abcam，Cambridge, UK | 1:1000 | 45 |
| BSP (DF7738) | Affinity, Cincinnati, OH, USA | 1:1000 | 36 |
| Runx2 (#12556) | Cell Signaling Technology，Danvers, MA, USA | 1:1000 | 60 |
| BMP2 (ab214821) | Abcam | 1:1000 | 44 |
| COL1 (WL0088) | WanLei, Shenyang, Liaoning, China | 1:1000 | 130 |
| ALP (ab108337) | Abcam | 1:10000 | 74 |
| GAPDH (10494-1-AP) | Proteintech, Chicago, IN, USA | 1:10000 | 36 |
